# Resistance and Virulence Features of *Bacteroides* spp. Isolated from Abdominal Infections in Romanian Patients

**DOI:** 10.3390/pathogens9110940

**Published:** 2020-11-12

**Authors:** Gabriela Roxana Pricop, Irina Gheorghe, Gratiela Gradisteanu Pircalabioru, Violeta Cristea, Marcela Popa, Luminita Marutescu, Mariana Carmen Chifiriuc, Grigore Mihaescu, Eugenia Bezirtzoglou

**Affiliations:** 1 Faculty of Biology, Department of Microbiology and Immunology, University of Bucharest, 060101 Bucharest, Romania; priroxana@yahoo.com (G.R.P.); dr.violetacristea@gmail.com (V.C.); lumi.marutescu@gmail.com (L.M.); carmen.chifiriuc@gmail.com (M.C.C.); mihaescuapelevii@gmail.com (G.M.); 2 Department of Earth, Environment and Life Sciences, The Research Institute of the University of Bucharest (ICUB), University of Bucharest, 050095 Bucharest, Romania; bmarcelica@yahoo.com; 3 Academy of Romanian Scientists, 050045 Bucharest, Romania; 4 Laboratory of Microbiology, Biotechnology and Hygiene, Department of Food Science and Technology, Faculty of Agricultural Development, Democritus University of Thrace, 67100 Orestiada, Greece; empezirt@yahoo.gr

**Keywords:** anaerobe, infection, *Bacteroides*, antibiotic resistance

## Abstract

Anaerobic bacteria are predominant residents of the normal microbiota of the skin and mucous membranes but are also known to be associated with a number of human infections including peritonitis, appendicitis, abscesses, ulcers and wound infections. Herein, we investigate the antibiotic resistance profiles as well as the genetic support of antibiotic resistance and virulence determinants of anaerobic bacteria isolated from intra-abdominal infections. The study was performed on 198 Romanian patients from which different clinical samples were taken intra-operatory and sent for microbiological analyses. From the total number of isolated strains, a subset of 75 *Bacteroides* spp. were selected and further investigated for antibiotic resistance and virulence features, at phenotypic and genetic level. Our results obtained through the analysis of a significant number of *Bacteroides* strains could shed light on the virulence potential and mechanisms by which anaerobic bacteria can cause endogenous infections.

## 1. Introduction

Anaerobic bacteria are predominant residents of the normal microbiota of the skin and mucous membranes in humans [1], but are also involved in a wide plethora of infections including peritonitis, appendicitis, odontogenic infections, cellulitis, abscesses, ulcers and wound infections [2].

Recent studies have shown a tremendous increase in the association of anaerobic bacteria belonging to *Bacteroides*, *Fusobacterium*, *Peptococcus*, *Propionibacterium*, *Bifidobacterium*, *Lactobacillus*, *Clostridium*, *Actinomyces*, *Finegoldia*, *Veillonella*, *Prevotella*, *Fusobacterium*, *Campylobacter*, *Porphyromonas* and *Peptostreptococcus* genera with clinical infections [3]. However, due to the fastidious nature of some species, anaerobic microorganisms are often difficult to isolate from infection sites and tend to be overlooked. Importantly, the polymicrobial associations and the emergence of antimicrobial resistance often complicate the treatment course of anaerobic infections. In clinical practice, infections caused by anaerobic bacteria are routinely treated using antibiotics such as carbapenems, metronidazole, beta-lactam/beta-lactamase inhibitor combinations, second generation cephalosporins, quinolones and clindamycin [3,4,5,6]. However, in recent years, increasing resistance rates have been reported worldwide especially for Bacteroides isolates. Indeed, Bacteroides strains are now almost completely resistant to tetracyclines and “regular beta lactams” (penicillins and cephalosporins) and moderately resistant to for moxifloxacin, clindamycin, cefoxitin and amoxicillin/clavulanic acid. A low level of resistance was shown in case of metronidazole, carbapenems, tigecycline, and piperacillin/tazobactam. The most frequent antibiotic resistance genes described to be associated with the main antibiotic resistance phenotypes in anaerobic bacteria are: cfiA or ccrA for carbapenems, cepA for “regular beta lactams” (penicillins and cephalosporins), cfx for cefoxitin resistance, *tetQ, tetM, tet36, tetX, tetX1* for tetracyclines *nim* genes (A-F), or the overexpression of *RecA* gene, or complete disruption of electron transport chain components are responsible for metronidazole resistance, bexA for quinolones and ermB, ermF, erm G, linA, the efflux pumps *msrSA* and *mefA* for the MLSB (macrolide, lincosamide, streptogramin B) group of antibiotics [7].

The aim of this study was to evaluate the antimicrobial resistance profiles and the corresponding resistance genes, as well as to highlight some virulence genes, in *Bacteroides* strains isolated from different intra-abdominal infections in patients hospitalized for abdominal surgery in Bucharest, Romania.

## 2. Results

### 2.1. Etiology of Intra-Abdominal Infections

A total of 374 bacterial strains were isolated from 198 patients with intra-abdominal infections submitted to abdominal surgery, during January 2015–February 2016. Most samples were harvested from peritoneal fluid (32%), appendix (15%), bile acid (13%), iliac pits (12%), abdominal abscess (8%), perforated ulcers (7%), perihepatic abscesses (5%), Douglas’ pouch (3%), blood culture (2%), mesentery (1%), pelvic abscesses (1%) and chronic gastritis (1%). Out of the tested clinical specimens, 171 (86.3%) samples were positive for either aerobic or anaerobic bacteria or mixed growth and the rest of 27 samples were negative. Among the 171 positive culture specimens, 27 (15.7%) showed only aerobic growth and 144 (72.7%) showed mixed anaerobic and aerobic growth.

From the total number of the isolated strains, 46% were represented by aerobic Gram-negative bacilli with *E. coli* (37%) being the most prevalent. The aerobic Gram-positive cocci, mostly comprised of *Enterococcus* species, represented 10% of the total number of the isolated strains (Table 1).

Among the anaerobic bacteria, the clinically significant isolates were represented by: *Bacteroides* spp. (20%), *Clostridium* spp. (8.2%) and *Parabacteroides* spp. (2%) (Table 2).

### 2.2. Antibiotic Resistance Profiles

As *Bacteroides* spp. represented the most prevalent etiology of anaerobic infections, these strains were further submitted to a more detailed analysis regarding their resistance and virulence profiles. These strains were isolated from different clinical specimens (i.e., from gallbladder, perihepatic abscess, wound infection and other intra-abdominal infections). From the total of 75 *Bacteroides* strains belonging to different species strains, 26 (35%) were *B. fragilis*, 16 (21%) *B. vulgatus*, 12 (16%) *B. uniformis*, eight (11%) *B. thetaiotaomicron*, seven (9%) *B. ovatus*, five (7%) *B. stercoris* and one (2%) strain of *B. caccae*.

All Bacteroides isolates were resistant to ampicillin (MIC 2 ≥ 16 mg/mL -), penicillin (MIC 2–4 mg/L) and tetracycline [MIC 8 - ≥8 mg/mL, according to Clinical Laboratory Standard Institute (CLSI) guidelines], 16 strains (21.9%) were resistant to clindamycin (MIC ≥ 8 mg/L), 8 (10.9%) to cefotetan (MIC 64 ≥ 64 mg/L) and only one strain (1.3%) to cefoxitin (MIC ≥ 32 mg/L). All strains were uniformly susceptible to metronidazole, augmentin, ampicillin-sulbactam, imipenem, meropenem, chloramphenicol and piperacillin with tazobactam. Concerning the antibiotic resistance profiles of different *Bacteroides* spp., 42.8% of *B. ovatus* strains showed resistance to cefotetan (MIC 32 ≥ 64 mg/L) followed by 37.5% of the *B. thetaiotaomicron* strains (MIC 8 ≥ 64 mg/L) and 6.25% of *B. vulgatus* isolates (MIC 4–64 mg/L) according to CLSI guidelines. The highest rate of clindamycin resistance (MIC 0.25 ≥ 8 mg/L) was harbored by *B. vulgatus* (56.2% mg/L) strains, followed by *B. uniformis* (8.3%) and *B. fragilis* (7.6%). The susceptibility testing results were interpreted according with CLSI and the European Committee on Antimicrobial Susceptibility Testing (EUCAST) guidelines (Tables S1 and S2).

### 2.3. Distribution of Resistance Genes among Bacteroides Isolates

**Figure 1:**
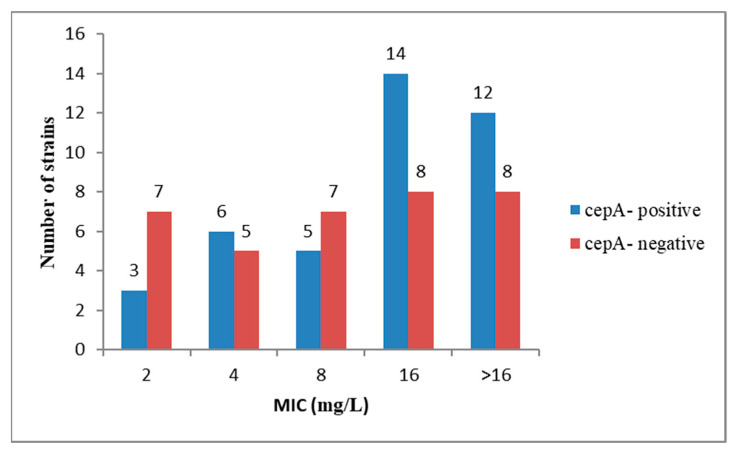
The presence of gene *cepA* was not correlated with ampicillin minimum inhibitory concentration (MIC) values.

**Figure 2:**
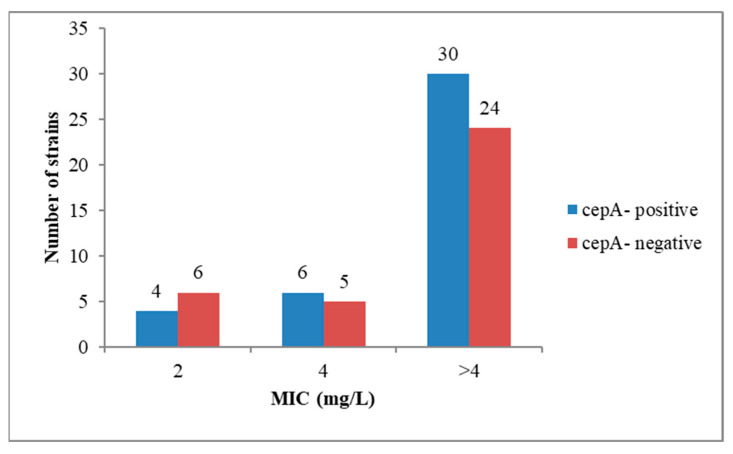
The presence of gene *cepA* was not correlated with penicillin MIC values.

Although all 75 *Bacteroides* spp. were resistant to ampicillin and penicillin, only 40 of them (53.3%) harbored the *cepA* gene and 55 (72%) strains were β-lactamase producers. The *cepA* gene was most frequently found amongst the *B. fragilis* strains; specifically, out of the 26 strains, 17 (65.3%) harbored the *cepA* gene. None of the *Bacteroides* strains was positive for *cfxA*, although one strain of *B. thetaiotaomicron* was resistant to cefoxitin (MIC ≥ 32 mg/L). It is to be mentioned that the presence of *cepA* gene was not correlated with the MIC value for ampicillin (AMP) and penicillin (PEN). Thus, out of the 20 *Bacteroides* strains with high ampicillin resistance (CMI ≥ 16 mg/L), only 12 were positive, while from the 54 strains that exhibited high penicillin resistance (CMI ≥ 4 mg/L), only 30 harbored the *cepA* gene (Figure 1 and Figure 2).

The lack of correlation between the presence of *cepA* gene and the MIC for AMP and PEN is suggesting the presence of other mechanisms of β-lactam resistance, such as mutations in the penicillin binding proteins (PBP) genes [8] or the presence of efflux pumps [9].

Of the 75 *Bacteroides* investigated strains, 16 (21.9%) were CLI resistant (MIC 0.25 ≥ 8 mg/L) and, in most cases, the resistant strains were positive for the *ermF* gene (18.6%) (Table 3 and Table 4). Several authors have demonstrated also the association between the presence of the *ermF* gene and the clindamycin resistance [10,11].

All strains were TET resistant (MIC 8 ≥ 8 mg/L) and, for the great majority of the investigated strains (98.6%), the resistance could be correlated with the presence of the *tetQ* gene (Table 3 and Table 4). This correlation was also reported in other studies by Meggersee and Abratt [12] and Szekely et al. [13].

### 2.4. Prevalence of Virulence Markers (VM) among Bacteroides spp.

Of the 75 investigated *Bacteroides* strains, 20 (26.6%) harbored the superoxide dismutase encoding gene (*sod)* involved in the pathogenicity of anaerobic bacteria, through its ability to neutralize the toxic levels of reactive oxygen species generated by the host [14]. A number of 13 (17.3%) *Bacteroides* strains were positive for the ferritin gene (*ftn*), involved in iron acquisition, and 8 (10.6%) were positive for the *katB* gene (catalase encoding gene, acting similarly with *sod* for decreasing the susceptibility of anaerobic bacteria to reactive oxygen species). The *bft* positive strains originated in the abdominal abscess and ascites fluid, followed by gallbladder and peritoneal liquid samples (Table 3).

Regarding the distribution of the *sod* gene by isolation sources, the gene was present mainly in the ascites fluid, peritoneal liquid and appendicular tissue samples. The majority of strains positive for the *ftn* gene were isolated from intra-abdominal abscesses (67%), whereas none of the strains from gallbladder harbored this gene. No strain isolated from abdominal abscess and perforated ulcer was positive for the *sod* gene.

Out of the total of 26 *B. fragilis*, 15 (57.6%) harbored the enterotoxin encoding gene (*bft*) (Table 3). *B. thetaiotaomicron* showed the highest percentage of *sod* (62.8%), followed by *B. fragilis* (34.6%), *B. ovatus* (14.2%), *B. uniformis* (16.6%) and *B. vulgatus* (12.5%). The *katB* gene was mostly identified in *B. fragilis* strains, which suggests the great survival ability of this species in the presence of oxygen.

## 3. Discussion

Our results in antibiotic resistance profiles are in accordance with Sarvari et al. [15], who recently reported a high level of resistance to AMP and TET in *Bacteroides* hospital-acquired strains, moderate level of resistance to CLI and susceptibility to MTR, AMC and CHL. Moreover, Javerica et al. [16] revealed also a high level of resistance to PEN in *Bacteroides* hospital-acquired strains.

Concerning the correlation between the level of resistance and the clinical origin of the *Bacteroides* spp., our results show that the gallbladder isolates harbored the highest number of antibiotic resistance genes, namely *tetQ* (100%), *cepA* (75%) and *ermF* (38%); while the strains isolated from perforated ulcers were the least resistant, presenting a lower frequency of *tetQ* (75%) and *cepA* genes (50%) (Table 3).

The *bft* positive strains originated in the abdominal abscess and ascites fluid, followed by gallbladder and peritoneal liquid samples, of which is different from other studies, where only 7.8% from peritoneal liquid strains were *bft* positive, a higher percentage of positivity was recorded for gallbladder strains (33.3%), and similar rates were reported for the appendix isolates (15.6% harbored *bft-1* gene) [17]. In our study of 26 *B. fragilis*, 15 (57.6%) harbored the enterotoxin encoding gene (*bft*). This positivity rate is higher than that reported in other studies (i.e., 13% [18], 18.46% [19] or 14.4%) [20]. When comparing these rates, it must be taken into account that the number of *B. fragilis* strains was lower in our study (26), compared to the cited ones (72/65), and the type of the analyzed samples and pathology was different (in our case, the 26 strains were isolated from patients with intra-abdominal infections).

## 4. Materials and Methods

### 4.1. Anaerobic Bacterial Strains Isolation and Identification

The study included 74 men (37.3%) and 124 women (62.6%) with the mean age of 55 years old, from which different clinical samples were taken intra-operatory and sent for microbiological analyses Synevo – Medicover Central Reference Laboratory, from Bucharest, Romania. The anaerobic strains were recovered following the cultivation of clinical samples in anaerobic conditions on anaerobic agar medium (Anaerobic Blood Agar, Phenylethyl alcohol agar and Schaedler Agar- Oxoid, Ireland, UK) and incubation at 37 ℃ for 2–7 days in GasPak pouch system (Becton Dickinson Microbiology System, Shannon Industrial Estate Shannon, County Clare, Ireland).

The strains identification was performed using matrix-assisted laser desorption and ionization time of flight mass spectrometry (MALDI-TOF) on the Byotyper platform (Bruker Daltonics, Bremen, Germany). The isolated strains were stored in BHI broth (brain heart infusion) with 20% glycerol at −80 ℃. From the total number of isolated strains, a subset of 75 *Bacteroides* spp. were selected and further investigated for antibiotic resistance and virulence features, at phenotypic and genetic levels.

### 4.2. Antibiotic Susceptibility Profiles

The antimicrobial susceptibility testing was performed using the microdilution method (Sensititre Anaerobe MIC Plate, TREK Diagnostic Systems, UK) according to the CLSI 2019 and EUCAST 2019 guidelines. Antibiotic susceptibility was tested for ampicillin-sulbactam (AMS), amoxicillin-clavulanic acid (AMC), cefotetan (CEF), penicillin (PEN), imipenem (IMP), meropenem (MEM), clindamycin (CLI), cefoxitin (FOX), metronidazole (MTR), chloramphenicol (CHL), ampicillin (AMP), tetracycline (AMP) and piperacillin-tazobactam (PIP-TZP) for all *Bacteroides* strains. The quality control strains used was *Bacteroides fragilis* ATCC 25285. For β-lactamase production, a chromogenic cephalosporin (OXOID, Basingstoke, Hampshire, UK) test was used.

### 4.3. Detection of the Antibiotic Resistance and Virulence Markers

The genetic support of antibiotic resistance (*cep*A, *cfx*, *tetQ* and *erm*F) and virulence (*bft-1, katB, sod* and *ftn* genes) was investigated by simplex and multiplex PCRs. Bacterial cells were resuspended in 20 µL solution 0.05 NaOH and 0.25% sodium dodecyl sulphate (SDS) and incubated at 95 ℃ for 15 min. Next, 180 µL TE (Tris EDTA) was added, followed by a 3 min centrifugation at 13,000 rpm. The supernatants were stored at −20 ℃. For PCR reactions, we used the primers described in Table 4.

The PCR reactions were performed using 2× PCR Master Mix (Dream Taq Green, Thermo Scientific, Waltham, Massachusetts, USA) in a final volume 20 µL. The amplicons were visualized by electrophoresis in 1% agarose gel, stained with ethidium bromide (10 µg/mL) and identified using molecular weight specific markers (100 bp, Thermo Scientific).

## 5. Conclusions

This study is among the few studies performed in Romania investigating the antibiotic resistance profiles as well as the genetic support of antibiotic resistance, and the first reporting on the virulence determinants of anaerobic bacteria isolated from intra-abdominal infections [23,24,25,26]. The bacterial etiology of intra-abdominal infections has a broad spectrum of bacterial species belonging to endogenous microbiota (Fam. *Enterobacteriaceae*, *Clostridium* spp. and *Bacteroides* spp.), with an incidence of anaerobic species of 40.3%. The species of *Bacteroides* genus dominated the anaerobic etiology, representing 49.6% of the total of 151 isolated anaerobic strains, followed by *Clostridium* sp. strains (20.5%). The *Bacteroides* strains were totally resistant to ampicillin, penicillin and tetracycline and exhibited variable resistance rates to clindamycin (21.9%), cefotetan (10.9%) and cefoxitin (1.3%). The isolated strains preserved entirely their susceptibility to metronidazole, amoxicillin/clavulanate, ampicillin with sulbactam, imipenem, meropenem, chloramphenicol and piperacillin with tazobactam. However, the frequency of antibiotics resistance/virulence determinants among analyzed strains could be higher if we take into account the entire anaerobic bacteria population of the sample. Due to the methodological restrictions assigned to the antibiotic susceptibility test (AST), where only a few colonies are picked up for AST, the result highlights only a small portion of the resistance reservoir. Studies show that when biological samples are incubated in the presence of different antibiotics, a surprising spectrum of resistant cells is revealed. This can be solved by changing the AST approach (i.e., replacing the selection of a few of colonies with a population-level approach), thus analyzing all bacterial population belonging to a clinical sample. This can be achieved by using new-generation PCR approaches which can measure in ~2–4 h the response of the entire bacterial population of a sample to different antibiotics [27,28,29]. The *Bacteroides* spp. exhibited different virulence genes profiles, depending on the isolation source, with the abdominal abscesses and ascites fluid isolates being the most virulent.

There is a paucity of scientific information regarding the genetic and molecular aspects of virulence factors during anaerobic intra-abdominal infections, thus our results, obtained through the analysis of a significant number of *Bacteroides* spp., could shed light on the virulence potential and mechanisms by which anaerobic bacteria can cause endogenous infections.

## Figures and Tables

**Table 1:** The prevalence of the aerobic bacteria in the analyzed clinical samples.

Group	Number	%
**Gram-negative bacilli**	**173**	**46**
*Escherichia coli*	140	37
*Klebsiella pneumoniae*	11	3
*Hafnia alvei*	13	3
*Proteus mirabilis*	6	2
*Morganella morganii*	3	1
**Gram-positive cocci**	**50**	**14**
*Enterococcus faecium*	24	7
*Enterococcus faecalis*	9	2
*Enterococcus durans*	5	1
*Streptococcus anginosus*	6	2
*Streptococcus constellatus*	6	2

**Table 2:** The prevalence of the anaerobic bacteria in the analyzed clinical samples.

Group	Number	%
**Gram-negative rods**	**94**	**25**
*B. fragilis group*	75	20
*Parabacteroides*	7	2
*Fusobacterium*	4	1
*Megamonas*	5	1
*Prevotella*	2	0.5
*Dialister*	1	0.2
**Gram-positive rods**	**44**	**12**
*Clostridium*	31	8.2
*Eggerthella*	2	0.5
*Cutibacterium*	11	2.9
**Gram-positive cocci**	**12**	**3**
*Finegoldia magna*	*7*	1.8
*Parvimonas*	5	1.3
**Gram-negative cocci**	**1**	**0.2**
*Veionella*	1	0.2

**Table 3:** The prevalence of the antibiotic resistance and virulence genes in the analyzed *Bacteroides* strains.

Species	Gene						
	(no.)%						
	*cepA*	*erm F*	*tet Q*	*bft-1*	*sod*	*katB*	*ftn*
***B. fragilis* (26)**	**17 (65.3)**	**4 (15.3)**	**26 (100)**	**15 (57.6)**	**9 (34.6)**	**7 (26.9)**	**7 (30.4)**
Peritoneal liquid	10 (32)	1 (3.2)	12 (38.7)	6 (19.3)	6 (19.3)	2 (6.4)	2 (6.4)
Intra-abdominal abscess	1 (33.3)	1 (33.3)	3 (100)	2 (66.6)	0	2 (66.6)	2 (66.6)
Appendicular tissue	2 (11.1)	0	3 (16.6)	2 (11.1)	1 (5.5)	1 (5.5)	0
Gallbladder secretion	3 (37.5)	0	3 (37.5)	2 (25)	1 (12.5)	0	0
Ascites fluid	1 (11.1)	2 (22.2)	4 (44.4)	3 (33.3)	1 (11.1)	2 (22.2)	2 (22.2)
Perforated ulcer-biopsy tissue	0	0	1 (25)	0	0	0	1 (25)
***B. vulgatus* (16)**	**10 (62.5)**	**4 (25)**	**15 (93.7)**	**0 (0)**	**2 (12.5)**	**0 (0)**	**0 (0)**
Peritoneal liquid	3 (9.6)	1 (3.2)	5 (16.1)	0	2 (6.4)	0	0
Intra-abdominal abscess	0	0	0	0	0	0	0
Appendicular tissue	3 (16.6)	0	3 (16.6)	0	0	0	0
Gallbladder secretion	2 (25)	3 (37.5)	4 (50)	0	0	0	0
Ascites fluid	1 (11.1)	0	1 (11.1)	0	0	0	0
Perforated ulcer-biopsy tissue	1 (25)	0	2 (25)	0	0	0	0
***B. uniformis* (12)**	**6 (50)**	**1 (8.3)**	**12 (100)**	**0 (0)**	**2 (16.6)**	**0 (0)**	**3 (25)**
Peritoneal liquid	0	0	3 (9.6)	0	0	0	1 (3.2)
Intra-abdominal abscess	0	0	0	0	0	0	0
Appendicular tissue	2 (11.1)	1 (5.5)	5 (27.7)	0	0	0	2 (11.1)
Gallbladder secretion	1 (12.5)	0	1 (12.5)	0	0	0	0
Ascites fluid	1 (11.1)	0	2 (22.2)	0	2 (22.2)	0	0
Perforated ulcer-biopsy tissue	2 (25)	0	1 (25)	0	0	0	0
***B. ovatus* (7)**	**1 (14.2)**	**1 (14.2)**	**7 (100)**	**0 (0)**	**1 (14.2)**	**0 (0)**	**2 (28.5)**
Peritoneal liquid	1 (3.2)	1 (3.2)	4 (13)	0	0	0	2 (6.4)
Intra-abdominal abscess	0	0	0	0	0	0	0
Appendicular tissue	0	0	3 (16.6)	0	1 (5.5)	0	0
Gallbladder secretion	0	0	0	0	0	0	0
Ascites fluid	0	0	0	0	0	0	0
Perforated ulcer-biopsy tissue	0	0	0	0	0	0	0
***B. thetaiotaomicron* (8)**	**4 (50)**	**3 (37.5)**	**8 (100)**	**0 (0)**	**5 (62.8)**	**1 (12.5)**	**1 (12.5)**
Peritoneal liquid	2 (6.4)	2 (6.4)	3 (9.6)	0	2 (6.4)	0	0
Intra-abdominal abscess	0	0	0	0	0	0	0
Appendicular tissue	2 (11.1)	1 (5.5)	4 (22.2)	0	2 (11.1)	1 (5.5)	1 (5.5)
Gallbladder secretion	0	0	0	0	0	0	0
Ascites fluid	0	0	1 (11.1)	0	1 (11.1)	0	0
Perforated ulcer-biopsy tissue	0	0	0	0	0	0	0
***B. caccae* (1)**	**1 (100)**	**0 (0)**	**1 (100)**	**0 (0)**	**1 (100)**	**0 (0)**	**0 (0)**
Peritoneal liquid	0	0	0	0	0	0	0
Intra-abdominal abscess	0	0	0	0	0	0	0
Appendicular tissue	0	0	0	0	0	0	0
Gallbladder secretion	0	0	0	0	0	0	0
Ascites fluid	1 (11.1)	0	1 (11.1)	0	1 (11.1)	0	0
Perforated ulcer-biopsy tissue	0	0	0	0	0	0	0
***B. stercoris* (5)**	**1 (20)**	**1 (20)**	**5 (100)**	**0 (0)**	**0 (0)**	**0 (0)**	**0 (0)**
Peritoneal liquid	1 (3.2)	1 (3.2)	4 (13)	0	0	0	0
Intra-abdominal abscess	0	0	0	0	0	0	0
Appendicular tissue	0	0	0	0	0	0	0
Gallbladder secretion	0	0	0	0	0	0	0
Ascites fluid	0	0	0	0	0	0	0
Perforated ulcer-biopsy tissue	0	0	0	0	0	0	0
**Total (no.)%**	**40 (53.3)**	**14 (18.6)**	**74 (98.6)**	**15 (57.6)**	**20 (26.6)**	**8 (10.6)**	**13 (17.3)**

no—number.

**Table 4:** Oligonucleotide primers used for the detection of virulence and resistance genes.

Gene	Primer	Size	Ref.
*bft-1*	5-GAGCCGAAGACGGTGTATGTGATTTGT-3 5-TGCTCAGCGCCCAGTATATGACCTAGT-3	500 bp	[21]
*katB*	5-GTAGCAGGAGAACGCGGAGCTGCT-3 5-GTTCATCCGCAGGCATCAGTCGGA-3	170 bp	GenBank
*sod*	5-ACAATGCGCTGGAACCTGTA-3 5-TTTCGAAGGTTTCGGAGCGA-3	230 bp	GenBank
*ftn*	5-ACG TTTCAGCGGTTTTGCAC-3 5-CGTTCGTGCTCAAAGACGTG-3	183 bp	GenBank
*cepA*	5-CGCAATGCCAAAGGACAACA-3 5-ACGATACGTGAGATGTCCGC-3	779 bp	GenBank
*tet Q*	5-CTGTTTGCCAGTGGAGCAAC-3 5-AGCAACTTTGTCTGCGCTTG-3	460 bp	GenBank
*cfxA*	5 -GCTCAAACAGATAGTTTTAT-3 5 -GAGCTCACAATGATGTTGCC-3	802 bp	[22]
*erm F*	5-AGGTGCAGGGAAAGGTCATT-3 5-ACCTCTGCCATTAACAGCAAT-3	446 bp	GenBank

## Supplementary Materials

The following are available online at https://www.mdpi.com/2076-0817/9/11/940/s1, Table S1: The antibiotic resistance profiles of the analyzed *Bacteroides* strains according CLSI 2019 (S = Susceptible; I = Intermediate; R = Resistant; R(%) = Resistance(%); Table S2 The antibiotic resistance profiles of the analyzed *Bacteroides* strains according EUCAST 2019 (S = Susceptible; I = Intermediate; R = Resistant; R(%) = Resistance(%).
