# Supplementary material for: Resistance and Virulence Features of Bacteroides spp. Isolated from Abdominal Infections in Romanian Patients
Source: Pathogens. 2020 Nov 12;9(11):940. doi: 10.3390/pathogens9110940 (PMC7696418; doi:10.3390/pathogens9110940)
Supplement: Supplementary file 1 [file pathogens-09-00940-s001.pdf]

Supplemental Table 1. The antibiotic resistance profiles of the analyzed *Bacteroides* strains according CLSI 2019(= Susceptible; = Intermediate; R=Resistant; R(% = Resistance(%)

**Antimicrobial**	**MIC breakpoint (µl/ml)**			***B. fragilis***		***B. vulgatus***		***B. uniformis***		***B. ovatus***		***B. thetaiotaomicron***		***B. caccae***		***B. stercoris***	
	**S**	**I**	**R**	**Range**	**R(%)**	**Range**	**R(%)**	**Range**	**R(%)**	**Range**	**R(%)**	**Range**	**R(%)**	**Range**	**R(%)**	**Range**	**R(%)**
Ampicillin	≤0.5	1	≥2	2 - ≥16	100	2 - ≥16	100	2 - ≥16	100	16 - ≥16	100	8 - ≥16	100	4	100	4-16	100
Amoxicillin-clavulanate	≤4/2	8/4	≥16/8	0.5/0.25-1/05	0	0.5/0.25-4/2	0	0.5/0.25-4/2	0	0.5/0.25-2/1	0	0.5/0.25-2/1	0	0.5/0.25	0	0.5/0.25-1/0.5	0
Ampicillin-sulbactam	≤8/4	16/8	≥32/16	0.5/0.25-2/1	0	1/0.5-4/2	0	0.5/0.25-2/1	0	1/0.5-2/1	0	0.5/0.25-2/1	0	1/0.5	0	0.5/0.25-2/1	0
Cefotetan	≤16	32	≥64	4-64	3.8	4-64	6.25	4-64	25	32 - ≥64	42.8	8 - ≥64	37.5	4	0	4-8	0
Penicillin	≤0.5	1	≥2	4 - ≥ 4	100	2- ≥4	100	2 -≥4	100	4 ->4	100	4 -≥4	100	4	100	2 -≥4	100
Imipenem	≤4	8	≥16	0.12 -0.25	0	0.12 -1	0	0.12 -25	0	0.25- 0.5	0	0.12 -0.5	0	0.12	0	0.12-0.5	0
Meropenem	≤4	8	≥16	0.5-1	0	0.5-2	0	0.5-1	0	0.5-1	0	0.5-1	0	0.5	0	0.5-1	0
Clindamycin	≤2	4	≥8	≥8	7.69	≥8	56.2	≥8	8.3	-≥8	28.5	≥8	12.5	0.25	0	0.25-0.5	0
Cefoxitin	≤16	32	≥64	4-16	0	2-16	0	2-16	0	4-16	0	4 - ≥32	12.5	4	0	1-2	0
Metronidazole	≤8	16	≥32	1-2	0	1-2	0	0.5-2	0	0.5-1	0	0.5-1	0	1	0	0.5-1	0
Chloramphenicol	≤8	16	≥32	2-8	0	2-8	0	2-4	0	2-4	0	2-4	0	4	0	2-4	0
Tetracycline	≤4	8	≥16	8 - ≥8	100	8 - ≥8	100	8 - ≥8	100	8 - ≥8	100	8 - ≥8	100	≥8	100	8 - ≥8	100
Piperacillin / tazobactam	32/4	64/4	≥128/4	0.25/4-2/4	0	1/4-4/4	0	0.25/4-0.5/4	0	0.25/4-2/4	0	0.25/4-2/4	0	1/4	0	1/4-2/4	0

Supplemental Table 2. The antibiotic resistance profiles of the analyzed *Bacteroides* strains according EUCAST 2019 (S = Susceptible; I = Intermediate; R = Resistant; R(%) = Resistance(%).

**Antimicrobial**	**MIC breakpoint (µl/ml)**		***B. fragilis***		***B. vulgatus***		***B. uniformis***		***B. ovatus***		***B. thetaiotaomicron***		***B. caccae***		***B. stercoris***	
	**S**	**R**	**Range**	**R(%)**	**Range**	**R(%)**	**Range**	**R(%)**	**Range**	**R(%)**	**Range**	**R(%)**	**Range**	**R(%)**	**Range**	**R(%)**
Ampicillin	≤0.5	≥2	2 - ≥16	100	2 - ≥16	100	2 - ≥16	100	16 - ≥16	100	8 - ≥>16	100	4	100	4-16	100
Amoxicillin-clavulanate	≤4	≥8	0.5/0.25-1/05	0	0.5/0.25-4/2	0	0.5/0.25-4/2	0	0.5/0.25-2/1	0	0.5/0.25-2/1	0	0.5/0.25	0	0.5/0.25-1/0.5	0
Ampicillin-sulbactam	≤4	≥8	0.5/0.25-2/1	0	1/0.5-4/2	0	0.5/0.25-2/1	0	1/0.5-2/1	0	0.5/0.25-2/1	0	1/0.5	0	0.5/0.25-2/1	0
Penicillin	≤0.25	≥0.5	4 - ≥4	100	2- ≥4	100	2 ->4	100	4 -≥4	100	4 -≥4	100	4	100	2 -≥4	100
Imipenem	≤2	≥4	0.12 -0.25	0	0.12 -1	0	0.12-25	0	0.25- 0.5	0	0.12 -0.5	0	0.12	0	0.12-0.5	0
Meropenem	≤2	≥8	0.5-1	0	0.5-2	0	0.5-1	0	0.5-1	0	0.5-1	0	0.5	0	0.5-1	0
Clindamycin	≤4	≥4	≥8	7.6	≥8	56.2	≥8	8.3	≥8	28.5	≥8	12.5	0.2	0	0.25-0.5	0
Metronidazole	≤4	≥4	1-2	0	1-2	0	0.5-2	0	0.5-1	0	0.5-1	0	1	0	0.5-1	0
Chloramphenicol	≤8	≥8	2-8	0	2-8	0	2-4	0	2-4	0	2-4	0	4	0	2-4	0
Piperacillin / tazobactam	≤8	≥16	0.25/4-2/4	0	1/4-4/4	0	0.25/4-0.5/4	0	0.25/4-2/4	0	0.25/4-2/4	0	1/4	0	1/4-2/4	0
